# Laboratory diagnosis of *Trypanosoma cruzi* infection: a narrative review

**DOI:** 10.3389/fpara.2023.1138375

**Published:** 2023-05-24

**Authors:** Constanza Lopez-Albizu, Rocío Rivero, Griselda Ballering, Hector Freilij, María Soledad Santini, Margarita María Catalina Bisio

**Affiliations:** ^1^ Departamento de Diagnóstico, Instituto Nacional de Parasitología (INP) "Dr. Mario Fatala Chaben", Administración Nacional de Laboratorios e Institutos de Salud (ANLIS) "Dr. Carlos G. Malbrán", Ministerio de Salud de la Nación, Buenos Aires, Argentina; ^2^ Departamento de Investigación, Instituto Nacional de Parasitología (INP) "Dr. Mario Fatala Chaben", Administración Nacional de Laboratorios e Institutos de Salud (ANLIS) "Dr. Carlos G. Malbrán", Ministerio de Salud de la Nación, Buenos Aires, Argentina; ^3^ Hospital de Niños “Dr. Ricardo Gutiérrez”, Buenos Aires, Argentina; ^4^ Consejo Nacional de Investigaciones Científicas y Técnicas (CONICET), Ministerio de Ciencia, Tecnología e Innovación de la Nación, Buenos Aires, Argentina; ^5^ Dirección, Instituto Nacional de Parasitología (INP) "Dr. Mario Fatala Chaben", Administración Nacional de Laboratorios e Institutos de Salud (ANLIS) "Dr. Carlos G. Malbrán", Ministerio de Salud de la Nación, Buenos Aires, Argentina

**Keywords:** Chagas disease, *Trypanosoma cruzi*, molecular diagnostic techniques, immunological tests, diagnostic reagent kits, parasitological diagnosis, neglected diseases

## Abstract

*Trypanosoma cruzi* infection, currently endemic in 21 countries, is a public health problem not only in the Americas but also in countries with Latin American migrants. However, it is estimated that two-thirds of people with Chagas disease currently live in urban areas and that only 10% of them are aware of it. This review summarizes the most important aspects of the diagnosis of human *T. cruzi* infection by describing the following aspects of clinical laboratory diagnosis: the most widely used tests available in Latin America and those expected to improve access to diagnosis of the affected population with their implementation; the advantages, disadvantages, and sensitivity of the tests in the different phases of infection; and their usefulness in the acute or chronic phases of infection and in the context of immunosuppression. In this way, we hope to contribute to broadening the knowledge about this prevalent infection in the Americas.

## Graphical Abstract

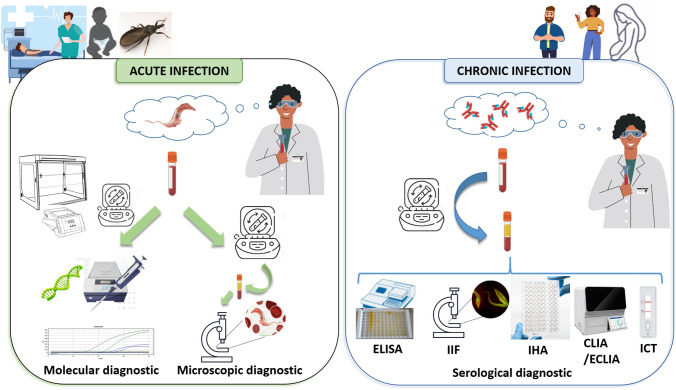

## 1 Introduction

**Figure 1:**
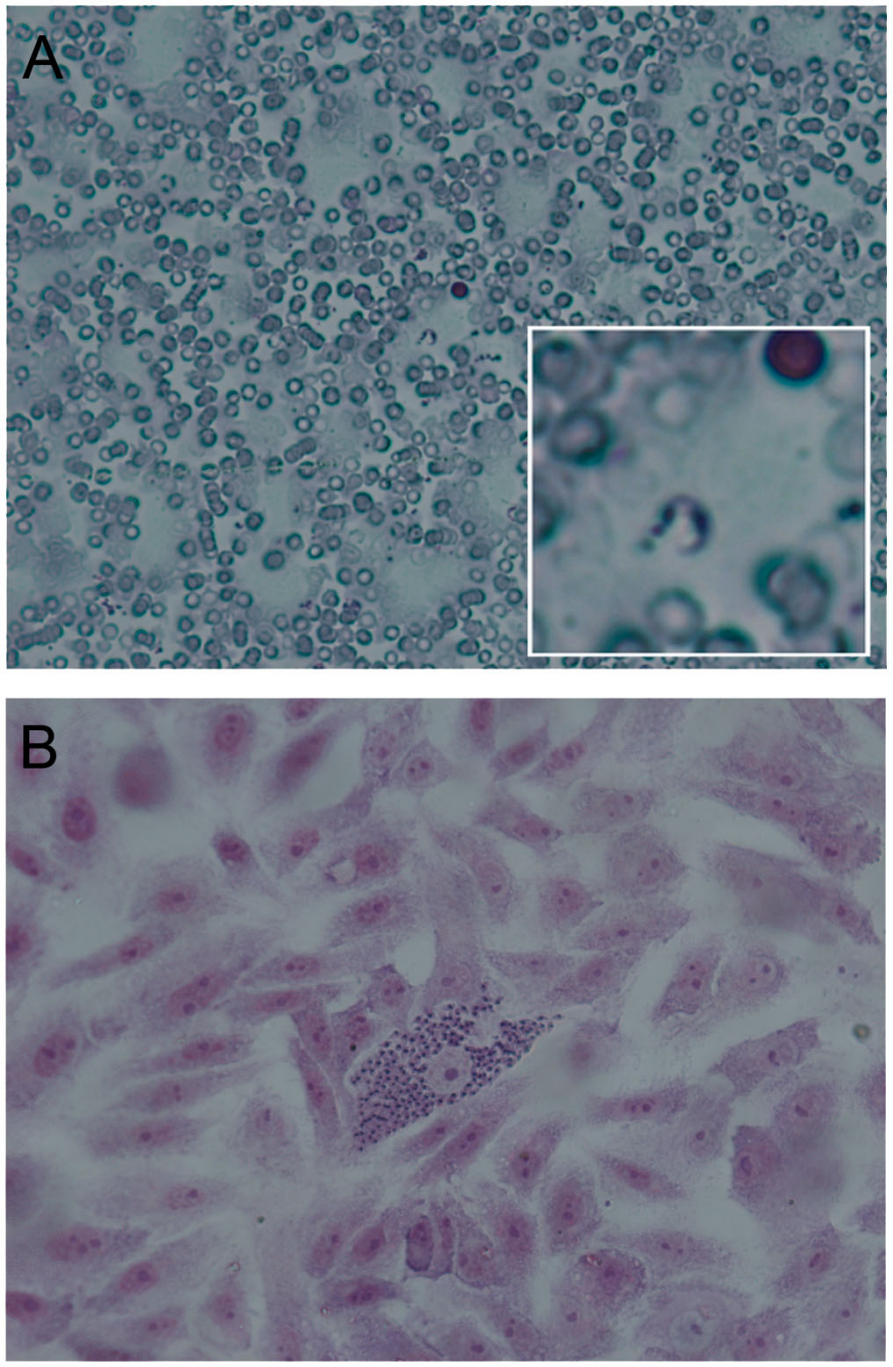
Stages of *Trypanosoma cruzi* in mammals. Hematoxylin-eosin staining, observation at 400X. **(A)** Trypomastigote: This form is elongated, with the kinetoplast located posterior to the nucleus. It is found in the blood of mammals and is the infective form. This form does not divide and is characterized by its mobility. **(B)** Nest of amastigotes: This form is spherical or oval and is the replicative form inside mammalian cells (mainly in muscle and nerve cells). This picture has been previously published [Lopez-Albizu et al. (2022)].

Chagas or American Trypanosomiasis is a parasitosis caused by the protozoan *Trypanosoma cruzi*. The distinguishing morphological feature is a prominent paraflagellar structure known as the kinetoplast, which corresponds to a condensation of DNA (kDNA) located within a single mitochondrion that is branched throughout the cell. During its biological cycle, which involves vertebrates and invertebrates, the parasite faces several environments and important changes during its interaction with the host cells. It presents three stages (epimastigote, trypomastigote and amastigote), which are defined based on cell morphology (pyriform, elongated or spherical) and on the relative position between the nucleus and the kinetoplast (anterior or posterior). The mammal infected by *T. cruzi* can present parasites either in the blood (in the trypomastigote stage, which is the extracellular infective form) or in tissues of different organs, mainly the heart, brain or digestive system (in the amastigote stage, which is the intracellular replicative form) (Storino Rubén and Milei, 1994) (**Figure 1**; Lopez-Albizu et al., 2022).

In some countries such as Colombia, Brazil, Peru, Costa Rica, Panama, Bolivia, Venezuela, Honduras, El Salvador, Mexico and Guatemala, the geographical range of *T. cruzi* overlaps with that of *T. rangeli* (Cuba Cuba, 1998; Grisard et al., 1999; Vallejo et al., 2009). This parasite is also transmitted by triatomine bugs and infects a wide variety of mammals, including humans. It shares both the triatomine vectors and mammalian hosts with *T. cruzi*, but is considered not pathogenic for the mammalian host and parasitemias tend to be quite low and transient. The biological cycles of both trypanosomatids are distinct. *T. rangeli* is pathogenic to its invertebrate host, while *T. cruzi* is pathogenic to its vertebrate host. Also, *T. rangeli* is transmitted through bites, as the infective forms are released in the saliva of the insect vector. In contrast, *T. cruzi* is typically transmitted through insect bites that cause skin lesions, or *via* the mucous membranes, as the parasite is eliminated in the feces of the vector (Cuba Cuba, 1998). Because of the similar geographical ranges, vectors, hosts and genotypes, and because of their genetic similarity, it is possible to confuse *T. rangeli* and *T. cruzi*. However, the blood stage trypomastigotes are morphologically distinct. *T. rangeli* is a longer parasite with a smaller kinetoplast, similar to African trypanosomes (Cuba Cuba, 1998; Guhl and Vallejo, 2003). In addition, it has been reported that 60% of the antigens purified by electrophoresis show cross-reactivity between both pathogens (Cuba Cuba, 1998; Grisard et al., 1999; Duffy et al., 2009). Therefore, for the serological diagnosis of *T. cruzi* infection in geographically overlapping areas, it is advisable to use recombinant antigens and synthetic peptides, and satellite DNA sequences instead of kDNA sequences for the molecular diagnosis (Cuba Cuba, 1998; Duffy et al., 2009; Duffy et al., 2013).

Based on the genetic differences within *T. cruzi* isolates, six discrete typing units (DTUs) (TcI to TcVI) have been defined (Zingales et al., 2009). In addition, a seventh lineage, named TcBat, which is mainly associated with bats, has been described (Marcili et al., 2009). However, the relationship of the infecting DTUs with the evolution of the infection and the development of specific clinical manifestations in humans continues to be a subject under study (Zingales, 2018).

Chagas is a health problem that has been included in the World Health Organization (WHO) list of neglected infectious diseases since 2005 (WHO, [[NoYear]]). It is endemic in 21 countries. The infection was originally limited to rural regions of the Americas, since the vectors that transmit *T. cruzi* live in the home and around the home of rural areas of the region. However, in the last decades, there has been an epidemiological change caused by: a) the implementation of strong vector control in rural areas, b) the migration of the population from the endemic countries of the Americas to the large cities of the region and even to other non-endemic countries, mainly the USA and Europe, and c) the shifting of the agricultural frontier and climate change. As a consequence, Chagas has become a global health problem, present not only in rural areas but also in large cities, and other routes of transmission with greater importance, such as vertical and oral routes, have emerged (WHO, 2015; Perez-Molina and Molina, 2018). This has also posed a challenge for diagnostic laboratories located in non-endemic countries.

The parasite is transmitted by different routes, among which vector transmission is the most important in endemic areas without vector control and vertical transmission in endemic areas where vector populations are under control, and in non-endemic areas (WHO, 2015; Perez-Molina and Molina, 2018).

Global estimates indicate that there are 70 million people at risk, between 6 and 7 million people infected, 30,000 new cases every year in the Americas alone, and 8,000 infants annually born with the infection. Moreover, 3 out of 10 infected people develop the disease and more than 12,000 people die from this disease every year (WHO, 2015; MSal, 2018). Thus, it is imperative to implement health strategies that ensure timely access to diagnosis, treatment, care, and follow-up for all people with Chagas disease who live in rural, peri-urban, and urban settings. Accurate, rapid, and timely diagnosis of *T. cruzi* infection represents a fundamental strategy in the approach to Chagas disease, since early treatment can prevent irreversible long-term consequences (PAHO, 2018). Treatment in childhood is highly effective and well tolerated, with few and mild adverse reactions (Sosa-Estani et al., 2012; PAHO, 2018). Furthermore, in recent years, there has been accumulating evidence that treating women of childbearing age is an intervention that can effectively reduce vertical transmission in future pregnancies (Sosa-Estani et al., 2009; Fabbro et al., 2014; Moscatelli et al., 2015; Alvarez et al., 2017; Murcia et al., 2017; MSal, 2018; PAHO, 2018).

Regarding the number of existing laboratory tests for the diagnosis of *T. cruzi* infection, the diversity of applied technologies, the scale of their use, and the heterogeneity of algorithms developed in different countries have increased rapidly in recent years. However, although different technologies and algorithms have been proposed in the scientific literature, they have not had a real impact in terms of public health. Indeed, in the Americas, coverage in the diagnosis of the infection continues to be deficient, and although it has grown in recent decades, it has not reached the expected objectives (Cucunuba et al., 2017; Klein et al., 2017; PAHO, 2017; Danesi et al., 2019).

This review summarizes the current status regarding the availability of laboratory tests for their clinical application in the diagnosis of *T. cruzi* infection, as well as recent developments, also providing a description of their advantages and disadvantages.

## 2 Direct and indirect microbiological diagnosis

In order to diagnose infectious diseases, fast, accurate, simple and accessible methods must be available. Microbiological diagnosis can be approached with various strategies, by making either a direct diagnosis (in which the etiological agent is identified) or an indirect diagnosis (in which the levels of specific antibodies against pathogen antigens are detected) (Washington, 1996).

**Figure 2:**
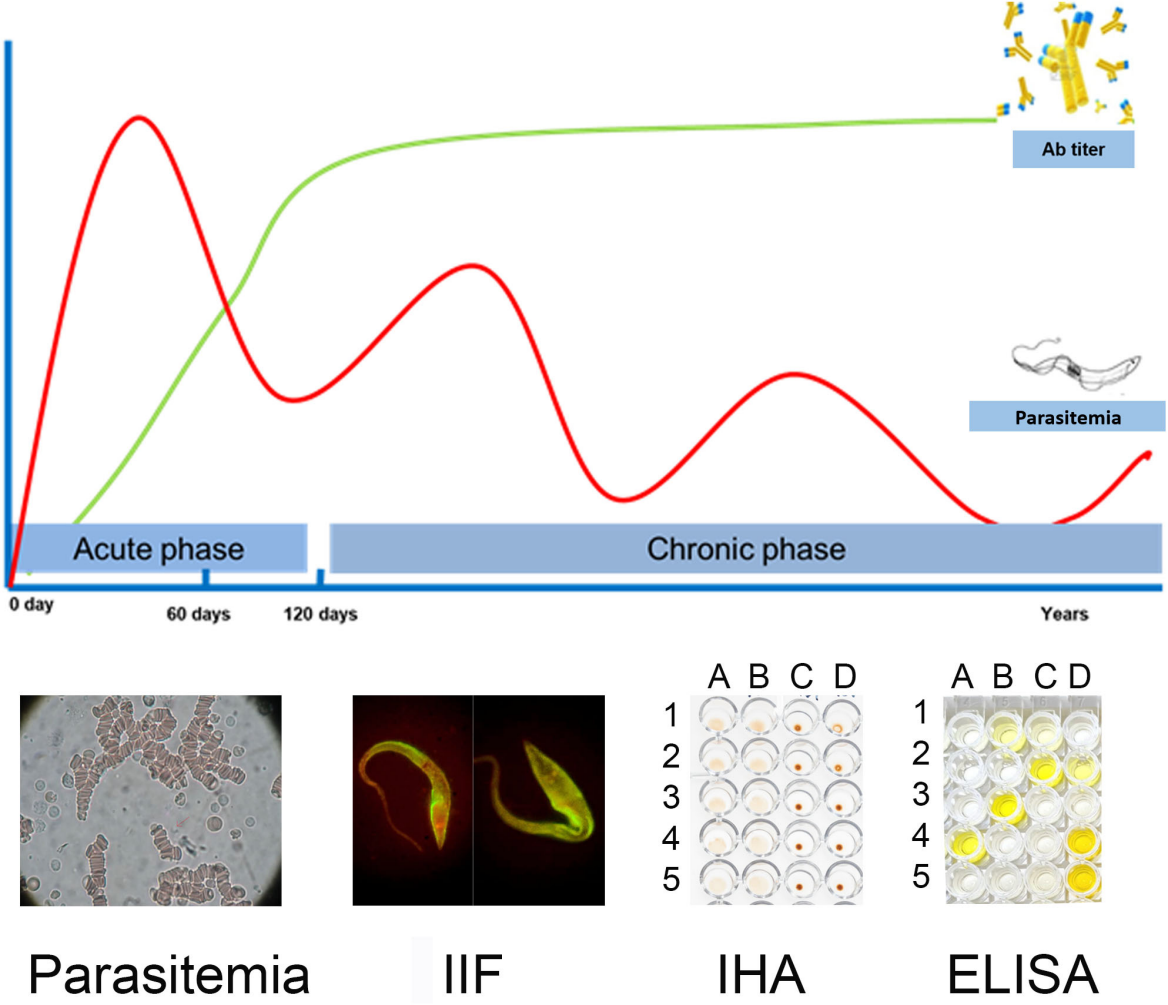
Schematic representation of parasitemia and specific anti-*T. cruzi* antibodies (Ab titer) in blood of infected patients during the course of infection. Some diagnostic tests used in each period of infection are shown as examples. Parasitemia: Microscopic image when performing the microhematocrit test in which the parasite is not seen but is illustrative of a preparation, since the search for the parasite is performed by observation of its movement, IIF: microscopic image of a preparation with serum from an infected subject, IHA: columns A, B: “sheaths” are observed as a result of the presence of anti-*T. cruzi* antibodies in the serum tested; columns C, D: “beads” are observed as a result of the absence of anti-*T. cruzi* antibodies in the serum tested. ELISA: image of an ELISA plate, showing the wells with positive results in yellow and negative results without staining. This picture has been previously published [Lopez-Albizu et al. (2022)].

*T. cruzi* infection has two phases: an acute phase and a chronic phase. In most of the patients, the acute phase lasts between 3 and 4 months from the entry of the parasite into the host, presents asymptomatically, and is characterized by a high load of circulating parasites in peripheral blood. This determines that the direct diagnosis should be implemented in this phase. The chronic phase begins when parasitemia decreases due to the patient’s immune response, and is characterized by the presence of IgG antibodies and low and intermittent parasite loads, detectable mainly by amplification methods (Rassi et al., 2012; MSal, 2018) This determines that the indirect diagnosis should be implemented in this phase. Thus, the natural course of the infection is what determines which microbiological diagnosis strategy is the most appropriate to implement [**Figure 2**, (Lopez-Albizu et al., 2022)].

### 2.1 Direct parasitological diagnosis

Qualitative parasitological tests commonly used in the laboratory diagnosis of acute *T. cruzi* infection or during infection reactivation in humans allow the detection of the parasite or any of its components. This section describes the direct methods, by first describing those aiming to detect the parasite by using microscopy (due to the advantage of the wide availability of the equipment required in clinical laboratories and to the possibility of obtaining an immediate result) and then those in which an amplification step is performed prior to detection (which show greater sensitivity but are only available in reference centers and whose result can take days or months).

#### 2.1.1 Microscopic detection

Direct observation under a microscope after concentration by centrifugation allows detecting the parasites alive due to their mobility. In situations of need and when there is no centrifuge available, the parasite can be detected using direct observation methods without prior centrifugation, by means of fresh drop (thin drop of blood between the slide and the coverslip) or thick drop (staining technique, also useful in areas where *T. cruzi* coexists with *Plasmodium*). However, for diagnostic purposes, tests that include a concentration step are recommended since this step increases the sensitivity of the test (Flores et al., 1966).

Depending on the volume of blood used, direct observation tests with a prior concentration step are called Strout or microhematocrit/micromethod. Parasites can also be observed in puncture fluids (trypomastigotes) as cerebrospinal fluid (CSF) or in biopsies (amastigote nests) [**Figures 1**, **2**, (Lopez-Albizu et al., 2022)].

##### 2.1.1.1 Strout’s test

The Strout’s test, first described in 1962 is the method recommended for the detection of parasitemia in adult patients with suspected acute infection or reactivation due to immunosuppression (Strout, 1962). It is performed by using a sample of 5 to 10 mL of venous blood collected in a tube without anticoagulant. It allows concentrating a greater amount of parasites with respect to the methods that use less volume. A study that compared the results obtained in 66 patients with suspected acute Chagas disease demonstrated a sensitivity of 61.8% of the Strout’s test versus xenodiagnosis (see below) (Flores et al., 1966).

##### 2.1.1.2 Microhematocrit and micromethod tests

The microhematocrit (or capillary) test, initiated by Worth, (1964), has been widely applied in the qualitative diagnosis of African trypanosomiasis (Woo, 1969). Subsequently, it was used by Freilij et al. in the search for *T. cruzi* (Freilij et al., 1983) and, since then, has been widely applied in the clinical setting for the diagnosis of vertical Chagas infection because it can be performed using a small sample volume (between 0.3 and 0.5 mL of blood) (La Fuente et al., 1984; Bittencourt, 1985; Freilij and Altcheh, 1995). To avoid the risk of cutting the glass of the capillaries and laboratory accidents, variants of the method have been optimized. These include: the rotation method, the oil immersion method, the wet mounting method by using capillaries, and the tube micromethod (Vera-Ku et al., 2019) and the micromethod using a 1.5-mL tube instead of a capillary (De Rissio et al., 2010). These tests have shown a sensitivity of 15-82% compared to serology at 10 months of life in the diagnosis of vertical infection (De Rissio et al., 2010; Velazquez et al., 2014; Messenger et al., 2017; Messenger and Bern, 2018; PAHO 2018; Bisio et al., 2021).

##### 2.1.1.3 Detection of *T. cruzi* in puncture fluids and biopsies

After the first report of encephalitis in a patient co-infected with *T. cruzi* and HIV in 1990 (Del Castillo et al., 1990) and with the increase in transplants and immunosuppressive therapies, detection of the parasite in other types of samples has become relevant for diagnosis and infection monitoring in immunodeficient patients. In addition to blood, parasites can be detected in pleural fluid, pericardial fluid, CSF, peritoneal fluid and biopsy samples of skin and other tissues (Ferreira et al., 1997; Iliovich et al., 1998; Sartori et al., 1999; Concetti et al., 2000; Lattes and Lasala, 2014). Regarding puncture liquids, they are centrifuged and the sediment is then observed between the slide and the coverslip. To detect nests of amastigotes in biopsies, histological sections are observed after staining with hematoxylin-eosin and Giemsa. The detection of parasites in biopsy transport fluid has been recently described (Lopez-Albizu et al., 2020a). No studies on the evaluation of analytical parameters or standardization have been carried out for this type of test. A systematic review of case reports of co-infection with HIV has described a sensitivity of 15% and 78% for the diagnosis of lesions at the central nervous system level in biopsy and CSF samples, respectively (Almeida et al., 2011).

##### 2.1.1.4 Advantages and disadvantages of microscopic detection

Microscopic detection tests have shown good results in reference centers, and high clinical sensitivity and specificity values for the diagnosis of vertical and acute Chagas disease. They have also been useful in the management of immunodeficient patients. They have the advantage of not requiring a highly complex laboratory and that the result can be obtained in less than 1 h after taking the sample.

However, these tests are carried out “in house” and some have not been subjected to analytical or clinical validation in prospective multicenter trials. Since the detection of the parasite depends on the observation of the mobility of live trypomastigotes, it has not been possible to implement external quality programs that allow the evaluation of the accuracy of the laboratories that perform it, ensure quality over time and make improvements (Bisio et al., 2021). Indeed, the sensitivity and specificity of these tests have mostly been evaluated in the context of referral centers and low performance has been described outside them (Flores et al., 1966; Freilij et al., 1983; Freilij and Altcheh, 1995; De Rissio et al., 2010; Velazquez et al., 2014). Therefore, they have traditionally been called “operator-dependent” tests, since clinical sensitivity depends on the operating conditions, the equipment available, and the experience of the personnel who observe the samples under the microscope.

#### 2.1.2 Detection of *T. cruzi* by amplification methods

Due to the low sensitivity of microscopic methods, parasitological and molecular amplification methods have been optimized. In addition to their high sensitivity for diagnosis in the acute phase, they can be used to try to demonstrate the presence of the parasite in the chronic phase (Flores et al., 1966; Chiari et al., 1989).

The parasitological methods of amplification are blood culture, inoculation in mice or by means of triatomines (xenodiagnosis). These methods have the disadvantage of requiring culture media, laboratory animals or vectors, trained personnel, long incubation times or development of the parasite cycle in the vector in blood culture or xenodiagnosis, respectively (2 months), biological safety cabinets, insectarium, birds to feed triatomines, vivarium, etc. Currently, these are not performed in clinical practice and are used only for research purposes, so they were not addressed in this review.

Molecular nucleic acid amplification methods began to be evaluated for their use in the diagnosis of human *T. cruzi* infection with the advent of polymerase chain reaction (PCR) in 1991 (Avila et al., 1991; Duffy et al., 2009; Benatar et al., 2021; Bisio et al., 2021). This first test (currently called endpoint PCR) has been used mostly in research and clinical trials. Its use has been extended to the clinical diagnostic field after the standardization of real-time PCR (qPCR) tests. On the other hand, as of 2010, new DNA amplification tests called isothermal tests have been developed. Two types of isothermal tests have been reported for *T. cruzi* infection: loop-mediated isothermal amplification (LAMP) and recombinase polymerase amplification (RPA), but only the former has been proposed for diagnostic use (Rivero et al., 2017; Besuschio et al., 2020; Ordonez et al., 2020).

##### 2.1.2.1 qPCR tests

**Figure 3:**
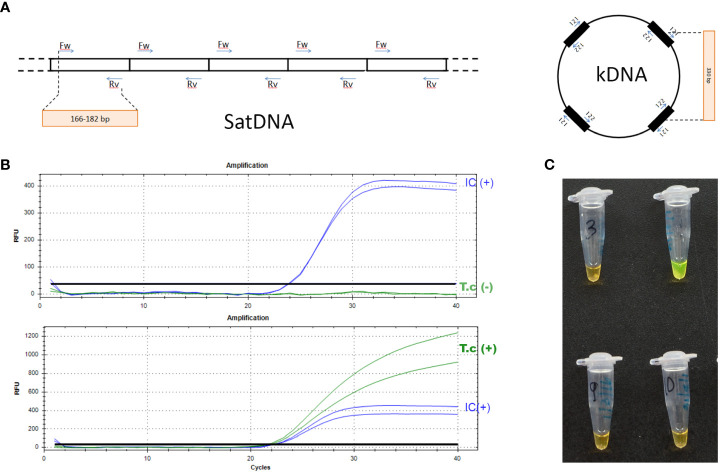
Nucleic acid amplification tests for diagnosis of *T. cruzi* infection. **(A)** Molecular targets used in DNA amplification reactions; **(B, C)** Visualization of laboratory results of real-time PCR and LAMP tests, respectively. SatDNA, nuclear satellite DNA sequences; kDNA, minicircle of kinetoplast DNA; T.c (+), detectable *T. cruzi* DNA amplification; T.c (–), not detectable *T. cruzi* DNA amplification; IC (+), detectable internal control amplification. This picture has been previously published [Lopez-Albizu et al. (2022)].

qPCR has been used in a large number of research studies and clinical trials for the diagnosis of *T. cruzi* infection, with promising results. Based on these results and those obtained in a multicenter study promoted by WHO and Pan American Health Organization (PAHO), the use of tests that amplify nuclear satellite DNA sequences (SatDNA) and kDNA is recommended (**Figure 3**) (Schijman et al., 2011; Duffy et al., 2013; Cura et al., 2017). Two in-house tests targeting SatDNA and kDNA sequences have been validated against the Clinical And Laboratory Standards Institute (CLSI) diagnostic standards; the reported analytical sensitivity was 0.70 parasites equivalents/mL (par eq/mL) and 0.23 par eq/mL respectively (Duffy et al., 2013; Ramirez et al., 2015). By using peripheral blood, a sensitivity of 84.2 to 100% was described for the diagnosis of vertical Chagas disease (Messenger et al., 2017; Bisio et al., 2021) and its use is also recommended in reactivations in the context of immunosuppression and post-treatment follow-up.

In recent years, at least eight commercial qPCR tests for *T. cruzi* infection have been developed (**Supplementary Table**). In 2021, a multicenter study was performed to evaluate the diagnostic performance of the prototype of one of these commercial kits, which amplifies SatDNA. The results of this study showed acceptable values of sensitivity and specificity for the diagnosis of vertical infection (Benatar et al., 2021).

##### 2.1.2.2 LAMP tests

In contrast to PCR tests, isothermal assays such as LAMP do not require equipment that cycles through temperature changes during the reaction (thermocyclers), or fluorescence readers, or software for data analysis, since the result is read by direct observation (color change) [**Figure 3C**, (Lopez-Albizu et al., 2022)]. This means that less equipment is required. After the first description of the use of LAMP for the detection of *T. cruzi* in feces of vector insects, different research groups have optimized it for the detection of *T. cruzi* DNA in humans (Thekisoe et al., 2010). However, this technique is still under evaluation for implementation. Two LAMP tests have been analytically validated (Rivero et al., 2017; Ordonez et al., 2020). One of them has been evaluated in a prospective study for its use in the diagnosis of vertical Chagas disease, with a performance similar to that of the microhematocrit test (Bisio et al., 2021). The other, a commercial kit prototype (Loopamp *T. cruzi* detection kit), has been evaluated using panels of human samples (Besuschio et al., 2017; Besuschio et al., 2020), but, to date, is not yet commercially available.

##### 2.1.2.3 Advantages and disadvantages of molecular nucleic acid amplification tests

Although PAHO has not included qPCR tests in the recommendations for the diagnosis of Chagas disease, in some countries, their use has been included in patient care guidelines. In Chile and Argentina, the use of qPCR has been recommended as an alternative to the direct parasitological method in the vertical Chagas diagnosis algorithm (MinSal, 2017; Lopez-Albizu et al., 2022; MSal, 2022). These tests have shown greater clinical sensitivity than microscopy for the diagnosis of vertical Chagas disease and are useful in the management of immunosuppressed patients. In addition, they allow automation, the preservation of samples for processing, the organization of the laboratory routines and the design of internal and external quality control programs by the reference centers to allow the continuous improvement. However, the supplies for molecular amplification tests are expensive and, in areas where there is no installed capacity to perform qPCR or LAMP, the samples must be derived, which means increasing their cost and a longer time between sample collection and delivery of the sample results with respect to microscopic tests.

### 2.2 Indirect serological diagnosis

Serological tests allow the detection of specific anti-*T. cruzi* circulating antibodies. Although some research studies have reported the detection of anti-*T. cruzi* IgM, this isotype is not commonly used for the diagnosis of acute infection. In contrast, there are multiple commercial tests for the detection of IgG, which are commonly used for diagnosis during the chronic phase of the infection [**Figure 2**, (Lopez-Albizu et al., 2022)] (Abras et al., 2016).

The first serological tests, usually called “conventional”, are based on semi-purified antigen lysates or fractions of *T. cruzi* epimastigotes obtained by culture (**Figure 5**). The disadvantages of conventional tests are that the occurrence of inconclusive, false-negative, and false-positive results has been persistently reported, and that there is no universally accepted reference standard (or “gold standard”) for the confirmation of results. The ideal serological test, with 100% specificity and 100% sensitivity, is unlikely to be achieved; however, it is possible to diagnose most cases using two serological tests (PAHO, 2018).

Currently, the antigens used for the detection of antibodies can be from whole cells, crude antigen from cell extracts or purified fractions, recombinant antigens and synthetic peptides (**Figure 5**) (Camargo et al., 1986; Houghton et al., 1999; Longhi et al., 2012; Bhattacharyya et al., 2014; Duarte et al., 2014; Abras et al., 2016). Tests based on recombinant antigens can be associated with surface proteins or cytoplasmic and/or flagellar antigens. However, since not all hosts produce antibodies against these antigens, the use of several recombinant antigens, ideally representing different stages of the parasite, could increase the sensitivity of antibody detection (Caicedo Diaz et al., 2019). In addition, differences in sensitivity have been observed between tests that use similar antigens but have different principles. These differences are mainly due to factors specific to the test that interfere or amplify the signal that expresses the antigen-antibody binding (Camargo et al., 1986). On the other hand, the diagnostic performance of the tests could be influenced by other factors such as regional differences in parasite antigenicity due to different DTUs. This has been suggested as an explanation for their limited performance in North and Central America (Duarte et al., 2014; Abras et al., 2016; Zingales, 2018; Caicedo Diaz et al., 2019; Kelly et al., 2021; Majeau et al., 2021). Since the results of serological tests are not generalizable to other geographical areas and the performance of the tests with different principles is potentially variable, the scientific evidence available is not sufficient to make changes in the current diagnostic algorithm. Therefore, PAHO indicates that *T. cruzi* diagnosis must be made through the “diagnostic standard” (MSal, 2018; PAHO, 2018), which considers that a person is infected when he/she has reactive results with two of the following pairs of tests of different principles and antigens: enzyme-linked immunosorbent assay (ELISA)-indirect agglutination assay (IHA), ELISA-immunofluorescence (IIF) or IHA-IIF. The two tests must be carried out in parallel and, in the event that only one is reactive, a third one that has not been used in the first processing should be added. Other tests based on principles of direct agglutination (such as latex agglutination test or direct agglutination, **Supplementary Table**) have been developed. Although they are used in laboratories, they have not been recommended by PAHO or WHO.

The serological methods that present greater specificity and have therefore been proposed as confirmatory tests are: radioimmunoprecipitation (RIPA), IIF and Western blot (WB) (Otani et al., 2009; Berrizbeitia, 2013; Brossas et al., 2021; Daltro et al., 2022). However, these methods are difficult to access for clinical diagnosis. Indeed, RIPA is only available in the USA and WB is only commercially available in Europe, so they will not be described in this article (**Supplementary Table**).

#### 2.2.1 Indirect hemagglutination assay: IHA

**Figure 4:**
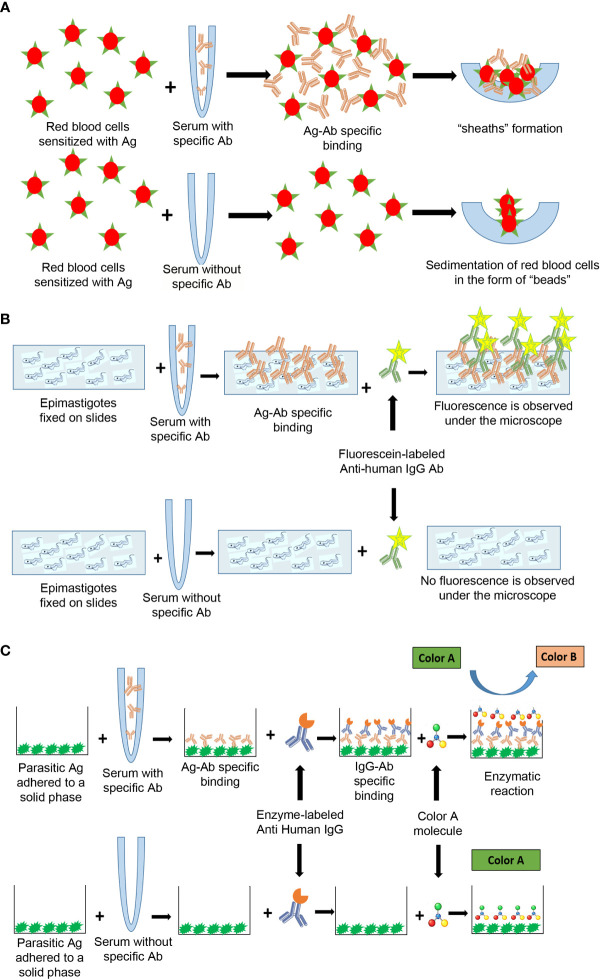
Antigen-antibody interactions in serological tests designed to detect anti-*T. cruzi* antibodies using different principles, **(A)** IHA, **(B)** IIF, **(C)** ELISA. Ag, antigen; Ab, antibody.

In the IHA, the antibodies present in the patient’s serum are detected by the formation of a network product of their interaction with the parasite’s antigens, which have previously bound to the sensitized membrane of the red blood cell. This network is visualized as a “sheath” or “thin film” at the bottom of the well where the reaction is carried out [**Figures 2**, **4**, (Lopez-Albizu et al., 2022)]. Serum is considered reactive for *T. cruzi* infection when this “sheath” covers more than 50% of the bottom of the well. If the antibodies are not present in the patient’s serum, the sensitized red blood cells settle in a “bead” at the bottom of the well [which covers less than 50% of the bottom of the well) (**Figures 2**, **4**, (Lopez-Albizu et al., 2022)].

This assay has the advantages of being inexpensive, easy to implement in laboratories (the reading of the results is made with the naked eye without the need for equipment), semi-quantitative, and useful in the follow-up of treated patients. A disadvantage is that if serial dilutions are not carried out, the prozone phenomenon and false negative results may occur (Linder and Miettinen, 1976).

#### 2.2.2 Indirect immunofluorescence: IIF

IIF uses the principle of primary antigen-antibody interaction (**Figure 4**). Cultured epimastigotes (antigens) are fixed to a slide and incubated with the serum sample. After washing, the slide is incubated with a secondary antibody, fluorescein-labeled anti-human IgG, which, in case of infection, recognizes the antigen-antibody complex (**Figure 4**). The preparation is observed under a fluorescence microscope. Fixed antigen slides are commercially available (**Supplementary Table**). The result is considered reactive if the fluorescence observed in the parasite’s membrane has greater intensity than that of the cytoplasm [**Figures 2**, **4**; (Lopez-Albizu et al., 2022)]. Among the tests with highest specificity, IIF is the most feasible to be implemented, due to its low cost and low complexity.

Although IIF is used in a large number of laboratories in Argentina and Latin America, it has the following disadvantages: i) a fluorescence microscope and trained personnel are needed to avoid the risk of erroneous readings (false positives) by interpreting sample reactivity when fluorescence is observed only in the cytoplasm of the parasite; and ii) like in the IHA, the prozone phenomenon may occur, for this reason, it is important to make serial dilutions (a minimum of three) in the serum sample (Linder and Miettinen, 1976; Mathai et al., 2020).

#### 2.2.3 Enzyme linked immunosorbent assay: ELISA

**Figure 5:**
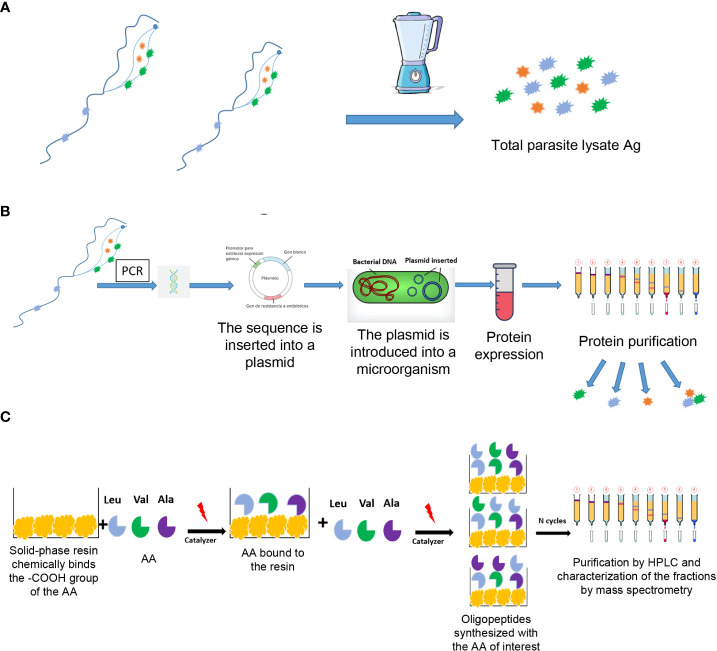
Different antigens used in the diagnostic serological tests designed to detect anti-*T. cruzi* antibodies. **(A)** Total extract (lysate) or purified antigens; **(B)** Recombinant antigens; **(C)** Synthetic peptides. Ag, antigen; AA, amino acid; HPLC, high performance liquid chromatography.

Commercial ELISAs are routinely used in clinical laboratories for the diagnosis of various infections. IgG-ELISA is a technique that has also been widely used for *T. cruzi* chronic infection. ELISAs uses the principle of primary antigen-antibody interaction. The antigens (lysate, recombinant or synthetic peptides) are fixed to a solid phase (plate) (**Figure 5**). Plates are incubated with a serum sample, followed sequentially by an enzyme-conjugated anti-human IgG and a substrate for the conjugate that is used. In case of infection, a color reaction will take place (**Figure 4**). There is a wide diversity of commercially available kits (**Supplementary Table**). Although the results can be read by direct observation, it is recommended to read the optical density or positivity ratio by using a spectrophotometer to accurately differentiate samples with absorbance values close to the cut-off value and report the optical density or the positivity ratio obtained. IgG ELISA is less subjective than IIF and IHA instead of immunofluorescence and large numbers of samples can be processed.

In addition to its usefulness as one of the tests within the serological pair for the diagnostic standard of chronic infection, at the end of the 2000s, a study was carried out to evaluate the different commercial ELISAs for the screening of donor blood. Based on the results of this study, and given the high sensitivity observed for these ELISAs, their use in blood banks is currently recommended (Otani et al., 2009; PAHO, 2018). This assay does not include confirmation of the diagnosis, but rather is a screening method to exclude potential positive donors.

#### 2.2.4 Electrochemiluminescence and chemiluminescence: ECLIA and CLIA

In recent years, automated equipment-based assays such as ECLIA and CLIA, used to diagnose other infections, have been developed for the detection of *T. cruzi* infection. These techniques consist in the use of soluble recombinant antigens marked with a chemiluminescent molecule and biotin, which binds to streptavidin. This technology has the capacity to process a large number of samples in a shorter period of time than manual tests. The greater sensitivity of ECLIA and CLIA, with respect to ELISAs, could be due to the fact that they use, in addition to a chemiluminescent or luminescent signal, a wide diversity of recombinant proteins which represent the three morphological stages and genetic diversity of the parasite (Abras et al., 2016; Flores-Chavez et al., 2018).

#### 2.2.5 Immunochromatographic tests: ICTs

Also known as lateral flow immunoassays (LFA) or rapid detection tests (RDT), ICTs allow obtaining the result in 30 minutes or less. They consist of specific antigens fixed on a nitrocellulose membrane. After sample addition, if there are specific antibodies against the fixed antigens, they subsequently bind to the antigen, developing a red line that can be directly observed.

These tests have the following advantages: they do not need equipment or refrigeration, the result can be obtained in minutes, and they can be performed at the point of care, without the need to refer a blood sample to the laboratory. However, in very hot regions, the manufacturer’s instructions regarding the maximum conservation and storage temperatures must be observed.

Although studies evaluating these ICTs have been conducted since the early 2000s, there are still no recommendations for their diagnostic use (Luquetti et al., 2003; Ponce et al., 2005; Roddy et al., 2008; Sosa-Estani et al., 2008; Chippaux et al., 2009; Verani et al., 2009; Lopez-Chejade et al., 2010; Barfield et al., 2011; Shah et al., 2014; Angheben et al., 2017; Eguez et al., 2017; Lozano et al., 2019; Mendicino et al., 2019; Lopez-Albizu et al., 2020b; Suescun-Carrero et al., 2021). In recent years, both the number of ICTs available on the market and clinical validation studies have increased (**Supplementary Table**). One difficulty in establishing recommendations for their implementation in diagnosis or screening is that few of these studies have used the diagnostic standard recommended by PAHO as a reference test (Luquetti et al., 2003; Shah et al., 2014; Mendicino et al., 2019; Lopez-Albizu et al., 2020b). On the other hand, some studies have evaluated the use of two ICTs as diagnostic tests (Eguez et al., 2017; Mendicino et al., 2019). Since, so far, there are no recommendations on their use for diagnosis, the positive results obtained during screening must be confirmed with the diagnostic standard (Sanchez-Camargo et al., 2014; Crudo et al., 2020; Lopez-Albizu et al., 2020b; Ortega-Arroyo et al., 2021).

## 3 Perspectives

The last 50 years have seen major advances in clinical laboratory diagnostic technology guiding high quality, safe and effective clinical decision making. These advances include the development of molecular diagnostics and recombinant antigens, the automation of serological tests, and the use of rapid point-of-care diagnostic tests. However, access to diagnosis of *T. cruzi* infection has not improved. Indeed, it is estimated that two-thirds of people with Chagas disease currently live in urban areas and that only 10% worldwide are aware of it (Sanmartino et al., 2015; Cucunuba et al., 2017; Klein et al., 2017; Danesi et al., 2019). As an example, PCR tests were rapidly implemented for the diagnosis of H1N1 Influenza virus and then, during the COVID-19 pandemic, for the detection of SARS-CoV2. However, it is paradigmatic that, although they have been used in multiple research studies since 1998 (Russomando et al., 1998), their implementation in Latin American diagnostic laboratories for the diagnosis of vertical Chagas disease has been slow and only Chile and, recently, Argentina have included them in their clinical recommendations (MinSal, 2017; MSal, 2022).

With the diagnostic tests currently available and the progress in quality evaluations and validations of the new technologies developed in recent years, it is possible to implement quality diagnostics for the early detection of *T. cruzi* infection, both in laboratories with limited resources and in highly complex centers that analyze a large number of samples daily (Rivero and Bisio, 2022). However, tools that allow greater access to diagnosis in primary healthcare settings are necessary. Although a wide variety of diagnostic tests have been described in both the scientific and the gray literature (more than 110 commercial tests for Chagas disease have been described, see **Supplementary Table**), there is still no gold standard. In particular, in recent years, many commercial kits have been developed, with their evaluation being partial and in different clinical-epidemiological contexts.

The present review describes the available diagnostic tests and provides a description from a narrative and conceptual perspective. An evidence-based evaluation of the performance of available immunoassays and nucleic acid amplification tests, which updates previously conducted systematic reviews and meta-analyses (Afonso et al., 2012), would facilitate decision-making regarding the purchase and implementation of these tests in the laboratory. Also, investment in the development, performance evaluation, and quality of diagnostic assays for vector-borne diseases and other poverty-related illnesses is scarce. The keys to achieving the sustainable development objectives set by international organizations are public awareness, motivation and training of health personnel, and political commitment at all levels.

## Author contributions

CL-A: literature review, Writing - original draft, Writing -review & editing. RR: Writing - original draft, conseptualization figures. GB: Writing - original draft. HF: Conceptualization, critical review of the manuscript MS: Conceptualization, Funding acquisition. MB: literature review, conceptualization, Writing - original draft, Writing - review & editing All authors contributed to review the final version of the article and approved the final version.
