# Supplementary material for: Laboratory diagnosis of Trypanosoma cruzi infection: a narrative review
Source: Front Parasitol. 2023 May 24;2:1138375. doi: 10.3389/fpara.2023.1138375 (PMC11732150; doi:10.3389/fpara.2023.1138375)
Supplement: Supplementary file 1 [file Table_1.docx]

Supplementary Material

Title: Laboratory diagnosis of *Trypanosoma cruzi* infection. A narrative review.

Constanza Lopez-Albizu, Rocío Rivero, Griselda Ballering, Hector Freilij, María Soledad Santini, Margarita María Catalina Bisio

*** Correspondence:** Corresponding Author: constanzalopezalbizu@gmail.com

# Supplementary Table: **List of commercial diagnostic tests for the serological detection of T. cruzi infection**

| **ID** | **Manufacturer** | **Test Name** | **Country Manufacturer** | **Antigen / Target** |
| --- | --- | --- | --- | --- |
| **Real-time PCR: qPCR** | | | | |
| 1 | Altona diagnostic | The RealStar® Chagas PCR Kit 1.0 | Germany | kDNA |
| 2 | BioManguinhos (Fiocruz) | ------ | Brazil | kDNA / SatDNA |
| 3 | CerTest Biotec | VIASURE Real Time PCR Detection Kit | Spain | SatDNA |
| 4 | Diagnositic Bioprobes | T. crzi DNA q PCR Kit | Italy | SatDNA |
| 5 | Genesing (pPrimerdesign) | T cruzi DNA Advanced Kit | United Kingdom | 18S |
| 6 | Progenie Molecular | RealCycler Chagas qPPCR Kit | Spain | SatDNA |
| 7 | Vivantis Technologies | ViPrimePlus T cruzi qPCR Kit | Malaysia | 18S |
| 8 | Wiener laboratorios | T. cruzi DNA q PCR Test | Argentina | SatDNA |
| **Loop-mediated isothermal amplification: LAMP** | | | | |
| 1 | FIND | Loopamp kit Chagas | Suiza | SatDNA |
| 2 | Laboratorio Cassará | Neokit Chagas | Argentina | Not available |
| **Indirect hemagglutination assay: IHA** | | | | |
| 1 | Biolab-Mérieux | Hemacruzi | Brazil | Bird erythrocytes sensitized with purified antigens |
| 2 | Ebram Produtos Laboratoriais Ltda | Chagas HAI | Brazil | Bird erythrocytes sensitized with *T. cruzi* |
| 3 | Gold Analisa Diagnóstica Ltda | Analisa HAI CHAGSAS | Brazil | Bird erythrocytes sensitized with purified antigens |
| 4 | Hemagen Diagnosticos Ltda | Chagas Hemagen HA | USA | Human erythrocytes sensitized with *T. cruzi* |
| 5 | Laboratório Lemos SRL | HAI Chagas Polychaco | Argentina | Sheep erythrocytes sensitized with *T. cruzi* |
| 6 | Wama Diagnostica | Imuno-HAI Chagas | Brazil | Bird erythrocytes sensitized with purified antigens |
| 7 | Wiener Lab | Chagatest IHA | Argentina | Sheep erythrocytes sensitized with cytoplasmic antigens |
| **Indirect immunofluorescence: IIF** | | | | |
| 1 | Biocientífica S.A | Inmunofluor Chagas kit | Argentina | * |
| 2 | Biocientífica S.A. (Inverness Medical) | Inmunofluor Chagas IFI | Argentina | * |
| 3 | Biolab Mérieux | IF Imunocruzi | Brazil | * |
| 4 | Bio-Manguinhos | IFI Doenca de Chagas | Brazil | * |
| 5 | Innogenetics Ibérica | IFA Kit Trypanosomiasis | Spain | * |
| 6 | MarDx Diagnostics, Inc. | MarDx IFA | USA | * |
| 7 | Tryniti-Mardx (Inverness Medical) | Kit Trypanosomiasis IFI | USA | * |
| 8 | Vircell | Chagas IFA | Spain | * |
| 9 | Wama Diagnostica | Imuno-com CHAGAS | Brazil | * |
| **Enzyme linked immunosorbent assay: ELISA** | | | | |
| 1 | Abbott Laboratories | Abbott ESA Chagas | USA | Recombinant antigens |
| 2 | Abbott Laboratories | Abbott PRISM Chagas | USA | Recombinant antigens |
| 3 | Abbott Laboratórios do Brasil Ltda | Abbott Chagas Anticorpos EIA | Brazil | Total extract (lysate) |
| 4 | Abcam | Anti-Chagas IgG ELISA Kit | United Kingdom | Not available |
| 5 | Adaltis | EIAgen *Trypanosoma cruzi* Ab | Italy | Total extract (lysate) |
| 6 | ATGen Diagnostica | CelQuest Chagas ELISA | Uruguay | Recombinant antigens |
| 7 | BIOKIT S.A. | Bioelisa Chagas | Spain | Recombinant antigens |
| 8 | Biolab-Mérieux | BioELISAcruzi | Brazil | Total extract (lysate) |
| 9 | BioMérieux | ELISA cruzi | France | Total extract (lysate) / purified antigens |
| 10 | Bio-Manguinhos | Bio-Manguinhos EIA | Brazil | Recombinant antigens |
| 11 | BIOSChile Ingenieria Genética S.A. | Test ELISA para Chagas III | Chile | Total extract (lysate) |
| 12 | BLK diagnostics | BLK | Spain | Total extract or purified antigens |
| 13 | Cellabs Pty Ltd. | *T. cruzi* IgG ELISA II | Australia | Recombinant antigens |
| 14 | Chemtest | CHEMLIS Chagas R-iELISA | Argentina | Recombinant antigens |
| 15 | Creative Diagnostics | Trypanosoma cruzi IgG ELISA kit | USA | Recombinant antigens |
| 16 | Demeditec Diagnostics GmbH | Chagas (Trypanosoma cruzi) IgG ELISA | Germany | Recombinant antigens |
| 17 | Diagnostic Automation/Cortez Diagnostics, Inc. | AccuDiag™ Chagas ELISA Kit | USA | synthetic peptides |
| 18 | Dia.Pro Diagnostic Bioprobes s.r.l | *T. cruzi* Ab (Chagas) | Italy | Recombinant antigens |
| 19 | DRG International Inc. | Chagas (Trypanosoma cruzi) IgG | Germany | Not available |
| 20 | Ebram Produtos Laboratoriais Ltda | Chagas ELISA | Brazil | Total extract (lysate) |
| 21 | EMBRABIO Empresa Brasileira de Tecnologia S.A | HBK 401 Hemobio Chagas | Brazil | Total extract (lysate) |
| 22 | Gador SA | Dia Kit Bio-Chagas | Argentina | Recombinant antigens |
| 23 | Hemagen Diagnósticos Ltda | Chagas Hemagen | USA | Purified antigens |
| 24 | IICS Univ de Asunción | Chagas Test IICS, ELISA | Paraguay | Total extract (lysate) |
| 25 | IVD Research Inc. | Chagas' Serum Microwell ELISA | USA | Not available |
| 26 | Laboratorio Lemos SRL | BIOZIMA Chagas | Argentina | Purified antigens |
| 27 | Laboratorio Lemos SRL | BIOZIMA Chagas Recombinante | Argentina | Recombinant antigens |
| 28 | Laboratorio Lemos SRL | Chagatek ELISA | Argentina | Purified antigens |
| 29 | Laboratorio Lemos SRL | Chagatek ELISA Recombinante | Argentina | Recombinant antigens |
| 30 | Laboratorio Pharmatest | Pharmatest Chagas | Venezuela | Not available |
| 31 | Meridian Diagnostics | Premier Chagas IgG ELISA | USA | Purified antigens |
| 32 | MyBiosource | *Chagas (Trypanosoma cruzi)* IgG ELISA Kit | USA | Total extract (lysate) |
| 33 | NovaTec Immundiagnostica | NovaLisa Chagas (*Trypanosoma cruzi*) IgG | Germany | Not available |
| 34 | Omega Diagnostics Ltd | Pathozyme Chagas | United Kingdom | Recombinant antigens |
| 35 | Orgenics | ImmunoComb II Chagas Ab | Israel | Recombinant antigens / synthetic peptides |
| 36 | Ortho-Clinical Diagnostics, Inc | ORTHO *T. cruzi* ELISA Test System | USA | Total extract (lysate) |
| 37 | REM Indústria e Comércio Ltda | Gold ELISA Chagas | Brazil | Recombinant antigens / purified antigens |
| 38 | Tecan IBL International GmbH | Chagas (*Trypanosoma cruzi*) IgG ELISA | Germany | Recombinant antigens |
| 39 | Tecnosuma Internacional | UMELISA Chagas | Spain | Synthetic peptides |
| 40 | Vircell | Chagas ELISA IgG + IgM | Spain | Recombinant antigens |
| 41 | Vircell | Chagas TESA ELISA IgG + IgM | Spain | Excretory-secretory antigens |
| 42 | Wama Diagnostica | Imuno-ELISA Chagas | Brazil | Recombinant antigens |
| 43 | Wiener Lab | Chagatest ELISA lisado | Argentina | Total extract (lysate) |
| 44a | Wiener Lab | Chagatest ELISA recombinante v.3.0 | Argentina | Recombinant antigens |
| 44b | Wiener Lab | Chagatest ELISA recombinante v.4.0 | Argentina | Recombinant antigens |
| **Electrochemiluminescence and chemiluminescence: ECLIA and CMIA** | | | | |
| 1 | Abbott Laboratories | Alinity i Chagas Reagent kit | España | Recombinant antigens |
| 2 | Abbott Laboratories | ArchitectC CHAGAS assay | España | Recombinant antigens |
| 3 | Biokit | BIO-FLASH Chagas | España | Recombinant antigens |
| 4 | COBAS, Roche Diagnostic | Elecsys Chagas | Alemania | Recombinant antigens |
| 5 | DiaSorin | LIAISON XL murex Chagas | Italia | Recombinant antigens |
| 6 | Vircell | Chagas Virclia | España | Recombinant antigens / purified antigens |
| **Immunochromatographic tests: ICTs** | | | | |
| 1 | AccuBiotech | Accu Tell Chagas Ab cassette | China | Not available |
| 2a | Acro Biotech, Inc. | Chagas Rapid Test Cassette - WB/S/P | USA | Recombinant antigens |
| 2b | Acro Biotech, Inc. | Chagas Rapid Test Cassette - S/P | USA | Recombinant antigens |
| 3 | Amunet | Amunet prueba rapida Chagas | Mexico | Not available |
| 4 | Atlas Link Technology | *T.cruzi* (Chagas) AB Test - Cassette | China | Not available |
| 5 | Beright | Cassette de Prueba Rápida de Chagas | China | Recombinant antigens |
| 6 | Bio-Manghinhos | Teste Rápido Chagas | Brazil | Recombinant antigens |
| 7 | Biozek | Chagas Rapid Test - Cassette | Netherlands | Not available |
| 8 | BTNX Inc. | The Rapid Response Chagas Antibody Test Cassette | Canada | Not available |
| 9 | Certum diagnostics | Prueba Rápida para Chagas en Cassette | Mexico | Recombinant antigens |
| 10 | Chembio | Chagas Stat-Pak Assay | USA | Recombinant antigens |
| 11 | Creative diagnostics | Chagas Ab Rapid Test | USA | Not available |
| 12 | CTK Biotech | OnSite Chagas Ab Rapid test | USA | Recombinant antigens |
| 13 | CTK Biotech | Chagas Ab Combo Rapid Test | USA | Recombinant antigens |
| 14 | Cypress Diagnostic | Chagas Quick Test | Belgium | Recombinant antigens |
| 15 | Diagnostic Automation/Cortez Diagnostics | Chagas Trypanosoma RapiDip | USA | Purified antigens |
| 16 | Encode Medical Engineering Co.,Ltd | Chagas Ab Combo Rapid Test | China | Not available |
| 17 | Hangzhou AllTest Biotech Co.,Ltd | Chagas Rapid Test Cassette | China | Recombinant antigens |
| 18 | HUMAN Diagnostics Worldwide | Hexagon Chagas | Germany | Recombinant antigens |
| 19a | InBios, Inc | Trypanosoma Detect Rapid Test | USA | Recombinant antigens |
| 19b | InBios, Inc. | Chagas Detect™ Plus Rapid Test | USA | Recombinant antigens |
| 20 | Innogenetics | INNO-LIA Chagas assay | Belgium | Recombinant antigens / synthetic peptides |
| 21 | Lemos laboratorios | Chagas Rapido First Response | Argentina | Recombinant antigens |
| 22 | Linear Chemicals S.L. | Chagas Ab cassette | Spain | Recombinant antigens |
| 23 | Meridian Biosciences | TruQuick™ Chagas 40T | USA | Recombinant antigens |
| 24 | Nal Von Minden GmbH | Test NADAL® Chagas IgG | Germany | Recombinant antigens |
| 25a | Operon | Simple Chagas WB | Spain | Recombinant antigens |
| 25b | Operon | Simple stick Chagas | Spain | Recombinant antigens |
| 26 | Quark Biotechnology Co., Ltd. | Chagas Ab Rapid Test | China | Recombinant antigens |
| 27 | Rapid Labs | Chagas Ab Rapid Test | United Kingdom | Not available |
| 28 | Span Biotech Ltd. | One-Step Chagas Ab Rapid Test | China | Recombinant antigens |
| 29 | Standard Diagnostic/Abbot | SD Chagas Ab Rapid | Korea | Recombinant antigens |
| 30 | Wiener Lab | WL Check Chagas | Argentina | Recombinant antigens |
| 31 | Xerion | Xerion Chagas Ac Combo | Colombia | Recombinant antigens |
| **Latex Agglutination Test** | | | | |
| 1 | DiaMed-ID Micro Typing System | ID-Chagas antibody test | Switzerland | Gel particles sensitized with synthetic peptides |
| 2 | Fujirebio, Inc. | Serodia Chagas | Japan | Gelatin particles sensitized with *T. cruzi* antigens |
| **Western blot: WB** | | | | |
| 1 | Abbott Laboratories | Abbot ESA Chagas | Estados Unidos | Recombinant antigens |
| 2 | Biolab Mérieux | TESA blot | Brasil | Excreted-secreted antigens |
| 3 | EMBRABIO Empresa Brasileira de Tecnologia S.A | HBK 740 IMUNOBLOT LINHAS anti-T.cruzi | Brasil | Recombinant antigens |
| 4 | LDBIO Diangostics | CHAGAS Western Blot IgG | Francia | Total extract |

kDNA,minicircle of kinetoplast DNA; SatDNA, satellite DNA sequences; 18s, 18S rDNA

*IIF use formalin-fixed epimastigotes
